# Analysis of mutational variations in *TP53* tumour suppressor gene among Pakistani head and neck cancer patients

**DOI:** 10.3332/ecancer.2024.1703

**Published:** 2024-05-16

**Authors:** Summera Fatima, Asia Bibi, Sara Samad Qureshi, Suman Khan

**Affiliations:** 1 Department of Zoology, The Women University Multan, Multan 60000, Pakistan; 2 Nishtar Medical University & Hospital, Multan 60000, Pakistan

**Keywords:** TP53, head and neck cancer, hotspot mutation, SSCP, missense mutation

## Abstract

The aim of this study was to determine the frequency of *TP53 *mutation among Pakistani head and neck cancer (HNC) patients who visited Nishtar Hospital Multan and Nishtar Institute of Dentistry (NID), Multan, Pakistan. While significant research has been conducted on the role of *p53 *in HNC throughout the world, this study is the first of its kind in Southern Punjab, Pakistan. A total of 242 samples (121 cases and 121 controls) were collected from Nishtar Hospital Multan and NID, Multan, Pakistan. After histopathological analysis to determine the stage type and grade of malignancy, DNA extraction and sequencing were carried out to assess any mutations in the *TP53* region (exons 5–8). Genetic screening was performed by the polymerase chain reaction (PCR)-single strand conformation polymorphism (SSCP) technique and Chromas 2.6.6 was used to visualise the sequencing results. The mean age of cases was 50.73 ±16.41 years and controls were 37.55 ± 15.51 years. The frequency of HNC was higher in male patients (65.28%) than in female patients (34.71%). Overall, this cancer was found to be significantly more prevalent in the age group of >35–55 years (*p* < 0.001). Smoking (51% versus 14%), naswar usage (15.7% versus 6.6%), poor oral hygiene (52.9% versus 29.8%) and anemic status (57.0% versus 4.1%) were significantly associated with cases (*p* ≤ 0.05) compared to controls. Only 04 samples exon 5 (1 sample), exon 7 (2 samples) and exon 8 (1 sample) with changed mobility patterns were found on the SSCP gel. All mutations were missense and heterozygous. Out of four mutant samples, three mutations were in the hotspot regions (codon 175, 245 and 248) of *p53.* Based on this study, there may be a weak association between the *TP53* exon 5–8 mutation and HNC patients in Southern Punjab, Pakistan.

## Introduction

Head and neck cancer (HNC) accounts for 5% of all cancers globally and is the seventh most common cancer worldwide [1–5]; however, in Pakistan, HNC accounts for 32.6% of all cancers [65] and is considered as second most usual cancer [65, 66]. HNC prevalence is expected to increase by 30% yearly in 2030 in both advanced and poor countries [3, 4, 6]. Its prevalence is different because of anatomical sub sites and geographical differences. Smoking-linked HNC has been found to be decreased in developed countries and a rising trend has been found in Human papillomavirus (HPV) linked HNC but in developing countries, a continuous rise has been seen [67–70]. The highest ratio of HNC has been recorded in South East Asia and some countries of Central and South Europe such as France and Belgium. The frequency of different types of HNC is high in Pakistan, India, France, Brazil, Bangladesh, Afghanistan, Nepal, Sri Lanka, Iran, Maldives and Bhutan [71, 72].

Major causes of HNC include hereditary and natural harmful components such as tobacco, Epstein-Barr Virus, HPV, arecanut, liquor, eating less carbs and other ecological and occupational exposures [7, 8]. Other harmful factors are ecological exposures to wool dust, wood dust, mineral filaments and low consumption of vegetables and fruits [9, 10]. The most common modifications in HNSCC are the disturbance in the pathway of *p53* [11, 12].

HNC is a complex illness that arises from changes in the tumour suppressor genes due to several gene-gene and gene-environment interactions. One of these genes is* TP53,* which is essential for controlling regular cell division and whose disturbance is the most frequent alteration in HNSCC (*TP53*; MIM#191170) [12]. In humans, the *TP53* gene encodes the transcription factor tumour suppressor *p53* protein. To maintain normal cellular functions and genome integrity, it is responsible for DNA repair, cellular homeostasis, metabolism, senescence and stimulation of non-apoptotic and apoptotic cell death. It is responsible for the activation of countless genes engaged in cell death metabolism and arrest of cell cycle in reaction to hypoxia, DNA damage, DNA mutations and oxidative stress, leading to stop the development of cancer [13]. So the loss of *p53* activity leads to gene deletion or mutation, results in failure of normal cell activity to regulate and repair DNA death or damage, and finally causes cancer due to genomic variability [73].

*TP53* mutations were confirmed in large-scale data in 2007 [14]. Almost 40%–60% of HNC patients have *TP53* mutations leading to cancer progression from premalignant to invasive form and early recurrence of the disease is increased in these patients [15]. Point mutation makes the *p53* protein non-functional or unstable, incapable of checking the proliferation of the cell. Non-functional *p53* protein increases due to overexpression of the gene *p53* and is reported in many studies [16]. Variations in *p53* are common and appear early in HNC and both tobacco carcinogenic agents and HPV disease, two major risk factors, appear to target *p53* [17]. The varying frequency (30% to 70%) of modifications in *TP53* HNC patients in different studies is due to HNC heterogeneity (like cancer of various organs) and various methods used to detect *TP53* mutational changes in HNC (such as mutational screening and immunohistochemistry). Many studies confirmed the role of *TP53* mutations with a frequency range of 75% to 85% in non-HPV-associated HNSCC patients [18–21]. The majority of *TP53* mutations shown by the International Agency for Research on Cancer affect exons 5–8, which make up the DNA-binding region specific to each site [22]. The goal of the current study was to determine if HNC patients from the Southern Punjab area had any mutations in the *TP53* gene exon 5–8.

## Materials and methods

In this study, we recruited a total of 242 participants, including 121 cases and 121 controls, from Nishtar Hospital Multan and Nishtar Institute of Dentistry, Multan, Pakistan, between 2017 and 2020. Informed consent was obtained from all the patients after informing them about the potential benefits of the study, in compliance with hospital rules. We collected Patient’s personal history, histopathological and other related information from the hospital and patients using a predesigned questionnaire. Ethical Approval for research work was obtained from the University Research Ethics Committee of Women University Multan (Reference No: WUM/UREC/00014).

The inclusion criteria for cases were HNC patients of any age (7 to 90 years), those not undergoing any treatment for cancer, and those with a clinical diagnosis of HNC. Fresh tissues and blood samples from 121 HNC cases were collected at the time of surgery before therapy. Various factors were compared between cases and controls including non-demographic factors (such as oral hygiene, diet, gum bleeding and anemic condition) and demographic factors (such as age, naswar use and smoking habit). The biopsy-proven HNC fresh tissues of any stage (TNM staging system includes tumour size and primary site, absence and presence of a metastatic condition of disease and nodal disease). The TNM stages are used to determine the overall stage of cancer, which ranges from 0 to IV [74], and were used in this investigation for the molecular analysis of the *TP53* gene. To preserve the tissue, it was immediately put in liquid nitrogen and stored at −80°C. Following the manufacturer’s instructions, genomic DNA was isolated from fresh, frozen tissues of HNC using Thermo Scientific DNA Purification (Cat # K0721). Sequencing was carried out following Single Strand Conformation Polymorphism analysis (SSCP). SSCP was used to find mutational alterations in the core DNA binding domain of the *TP53* gene, which contains important hot spots. Exon 5–8 regions of *P53* were selected that give amplicons of sizes ranging from 180–247 base pairs. A mutant forward primer with a single nucleotide alteration by replacing T with G was chosen to provide the mutant positive control of exons 5 and 8. A DNA sequence altered from A to G was chosen as a mutation-positive control for exon 6, and a DNA sequence changed from G to C was chosen as a mutation-positive control for exon 7. Sequencing was used for the confirmation of all these mutant controls.

PCR cycling conditions were set at 95°C for 5 minutes, followed by 30 cycles of 30 seconds at 95°C, 45 seconds at the annealing temperature of the primers used (Exon 5 = 57°C, Exon 6 = 54°C, Exon 7 = 59°C and Exon 8 = 56°C), and 30 seconds at 72°C using the thermal cycler G-Storm 4822. The gel electrophoresis was conducted in 1× TAE buffer for 30 to 45 minutes at 100V followed by visualisation and photography using an ultraviolet transilluminator.

When Exon 5–8 of the *TP53* gene were amplified then SSCP analysis was carried out on the Decode™ Universal Mutation Detection System, Biorad (Cat. # 170-9092). For the optimisation of SSCP of all four exons and gels were run in varying conditions, i.e., a. Exon 5 and 6: 60 mL of 12% non-denaturing polyacrylamide gel solution was prepared by mixing 24 mL of 30% acrylamide mix (29:1 acrylamide/bisacrylamide), 6 mL of 10× Tris boric acid EDTA (TBE) (pH 8.3) and 30 mL of deionized water and stored at 4°C. b. Exon 7 and 8: 60 mL of 12% non-denaturing polyacylamide gel solution was prepared by mixing 24 mL of 30% acrylamide mix (29:1 acrylamide/bisacrylamide), 33 mL of 10× TBE (pH 8.3) and 30 mL of deionized water and stored at 4°C. 

For SSCP analysis, 5 µL PCR product was mixed with 15 µL of loading buffer (0.5 M NaOH, 0.05% bromophenol blue, 0.05% xylene cyanol and 50% (*w*/*v*) Sucrose) denatured at 96°C for 8 minutes and immediately placed on ice. Twenty microliters of each denatured PCR product (containing 30–40 ng DNA) were loaded onto a 12% gel. Electrophoresis was conducted with TBE running buffer (1× for exon 5 and 6: 0.5× for exon 7 and 8) at 220V for 10–12 hours until xylene cyanol reached the bottom of the gel. The temperature for electrophoresis was 20°C for exon 5 but for exon 6, 7 and 8, the best results were found at 4°C. After electrophoresis, the gels were separated from the plates using a gel separator and transferred to a tank containing an ethidium bromide solution (250 mL of deionized water and 250 uL of 10 mg/mL ethidium bromide solution) for 5 to 10 minutes. The gel was visualised on a UV transilluminator and photographed.

For results interpretation, we compared the SSCP band patterns of normal control, mutant control DNA and cases. No mutations were observed in control samples. Confirmation of all mutation-positive cases was done at least three times by a separate SSCP run and PCR reaction. Control samples did not show any mutation. Only those samples that exhibited a changed pattern in the form of extra bands and mobility shifts with respect to normal samples have proceeded for sequencing. These PCR products were amplified under the appropriate conditions, run on a 1.5% agarose gel and the bands exhibiting a changed pattern were cut to isolate DNA for sequencing using a Thermo Scientific GeneJET Gel Extraction kit (Cat. # K0691). Sequencing analysis was performed using the Sanger sequencing method and chromatograms were used to record sequencing results. The chromatograms were used to visually analyse the data and identify changes in the human *TP53* gene in HNC patients.

The SPSS software, (version 20) was used to conduct statistical analyses. The demographic and non-demographic characteristics of cases and controls were assessed by the *χ*^2^ test. Multiple logistic regression was applied for multivariate analyses. We considered *p*-values of ≤0.05 as statistically significant.

## Results

Out of the total 242 study participants, there was no difference in gender between the cases and controls, with an equal number of males (*n* = 79) and females (*n* = 42) in both groups. The mean age of the study population, ranged from 7 to 90 years, with cases having a mean age of 50.73 ± 16.41 years and controls having a mean age of 37.55 ± 15.51 years. A statistically significant difference (*p* ≤ 0.05) was observed among the groups with reference to demographic factors. HNC patients were significantly more frequent in the age group of >35–55 years (*p* < 0.001). HNC patients showed a high frequency of smoking (51% versus14%; *p* < 0.001), smoking frequency >10 times/day (90% versus 55%; *p* < 0.001), smoking duration >15 years (74% versus 5%; *p* < 0.001), naswar usage (15% versus 6%; *p* = 0.029), naswar use frequency 6–10 times/day (68% versus 50%; *p* = 0.370) and naswar use duration >20 years (52% versus 12%; *p* = 0.069) as compared to controls (Table 1).

Poor oral hygiene condition was significantly associated with cases (52% versus 29%; *p* = 0.026) as compared to controls but there was no significant association (57% versus 5%; *p* = 0.055), regarding gum bleeding factor between cases and controls. Anemic status was highly significantly associated with cases (57% versus 4%; *p* < 0.001) as compared to controls. With respect to diet, meat fonder was more frequent in cases as compared to controls (12% versus 3%; *p* = 0.055) (Table 2).

**Figure 1.**
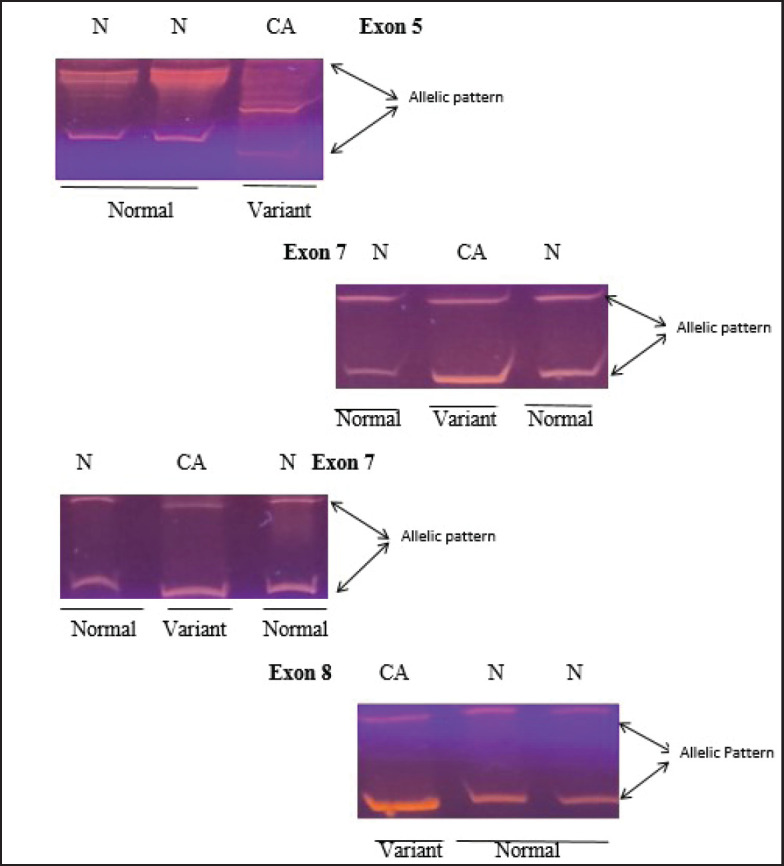
SSCP analysis of normal and variant mobility pattern. CA represents HNC patient while N indicates normal control gene amplified for exons 5, 7, and 8 of *p53* gene.

**Figure 2.**
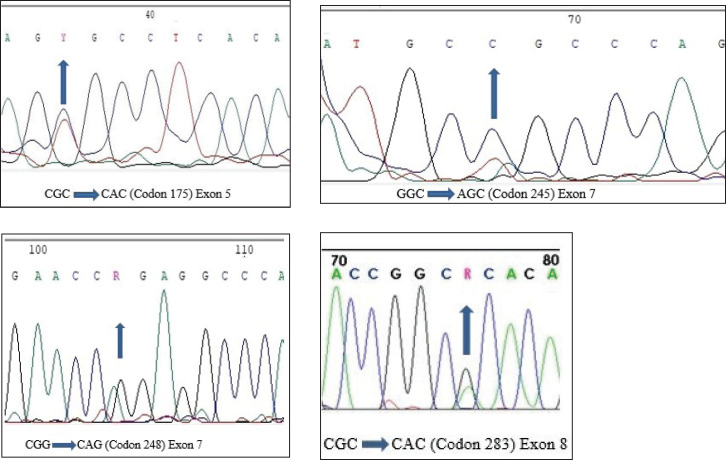
DNA sequencing chromatogram representing heterozygous mutations in each of exons 5, 7 and 8.

**Figure 3.**
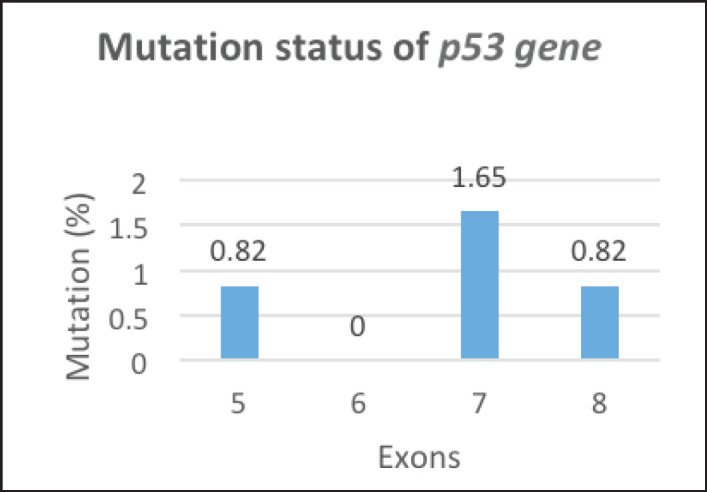
Mutation status of *p53* gene.

Only f4 out of 121 HNC samples (Exon 5, 7 and Exon 8) showed changed mobility patterns on the SSCP gel (Figure 1). All four mutant samples were from males. The affected site in Exon 5 and 7 was the larynx in three males, while in one male, the affected site (Exon 8) was the oral cavity. The patients had different occupations, with three being farmers and one being a cook. Tumour grade 2 was common in all four patients and all patients with the mutation had tumour stage III, with the affected site being the larynx, except for one patient who had tumour stage II, with the affected site being the oral cavity. Three of the patients smoked at the frequency of 20 times per day. Among the patients showing mutation, one was 45 years old and three patients were 60 years old (Table 3). Figure 2 shows the DNA sequencing chromatogram of mutations in Exon 5, 7 and 8. All exons sequencing chromatograms showed heterozygous mutations. In all four mutant samples, a missense mutation was observed. In Exon 5, a CGC > CAC (Arg > His) mutation was observed, and in Exon 7, a CGG > CAG (Gly > Ser) and CGG > CAG (Arg > Gln) mutations were observed and in Exon 8 a CGC > CAC (Arg > His) mutation was found. All four mutant samples had transition mutations. Mutations were found in the following codons; codon 175 (Exon 5), 245, 248 (Exon 7) and 283 (Exon 8) of the *p53* gene (Table 4). The percentage of *p53* mutation in Exon 5 and 8 was 0.82% and in Exon 7, it was 1.65%, while in Exon 6, it was 0% (Figure 3).

## Discussion

In this study, we compared demographic factors and non-demographic factors between cases and controls. The mean age of cases was 50.73(±16.41) years and controls were 37.55(±15.51) years. Similar mean age ranges were observed in different studies [23, 24], while in contrast, a mean age of 63.8 ± 9.3 years was found in both cases and control in a 2019 study [25]. A significant difference in demographic features was observed between cases and controls, which is consistent with one Pakistani study [26], while a contrary result was found in another Pakistani study [24]. Regarding smoking, the number of smokers was higher in cases compared to controls, as found in different studies [24, 27], but contrary to other studies [26, 25] where the frequency of smokers was higher in controls. In the current study, smoking frequency (>10 times/day) was more in cases than in controls, as found in a study conducted in 2020 [27]. Additionally, we observed a significant association in the case of naswar usage between cases and controls. The frequency of naswar use was higher in cases than in controls in this study, similar to a Pakistani study [24]. Therefore, naswar may be one of the risk factors for HNC, which is in agreement with one Pakistani study [28].

Oral health is crucial for overall health and well-being. Poor oral health can lead to various oral diseases. In this study, poor oral health conditions were more prevalent in cases than controls, consistent with other studies [29, 24].

Due to low literacy rates and limited income, people in this area neglect their oral health. Gum bleeding was observed more frequently in cases in line with other studies [30, 31, 23]. Our study found a significant association between cases and controls regarding anemic status and diet. The percentage of anemic patients was higher in cases as reported in one study [32] and more vegetables were consumed by cases than controls consistent with other studies [33, 34]. However, unlike our study, some studies have reported an inverse relationship between vegetable consumption and HNC [35–37]. Additionally, our study found that cases consumed less meat, whereas studies conducted in Asia reported an inverse relationship between red meat consumption and HNC risk [38].

In this study, the frequency of *TP53* mutations was low (3.3%) which is similar to the findings of many studies [39–43] where it was 3.2%, 3%, 2%, 3.4% and 0%, respectively. However other studies have reported a higher frequency of *TP53* mutations [44–48], where it was 24%, 25%, 21.5%, 16% and 66.2%, respectively. The reasons for the variation in *TP53* mutation frequency observed in different studies may be due to differences in detection techniques and the number of exons (2–11 exons) analysed. Therefore, it is more challenging to draw conclusions that can be applied across studies from a comprehensive evaluation of the literature [46, 49, 50]. Nonetheless, our study supports the notion that the incidence of *TP53* gene mutations differs globally, even within the same country.

In the current study, three mutant samples had affected site larynx (Exon 5 and 7) similar to different studies [51, 8] and one had affected site oral cavity (Exon 8), consistent with the study of Peltonen *et al* [51]. 75% of mutations were found in hotspot codons of *p53,* i.e., 175, 245 and 248 codons, as found in different studies [45, 51, 8, 52, 53]. No mutation was found in Exon 6 contrary to the finding of Peltonen *et al* [51]. All mutations in the *p53* gene found were missense mutations similar to different studies [51, 54, 8, 52, 55] that reported 90% of mutations in the *p53* gene as missense mutations.

In the current study, all exon sequence chromatograms showed heterozygous mutations similar to the study of Leng *et al* [56] and contrary to the study of Baugh *et al* [52] which reported homosygous mutations in 50% to 60% of human cancer. Mutation in codon 245 was found in Exon 7, which matches with different studies [45, 58, 57, 8] that reported it as the most prevalent hotspot codon in HNC. The percentage of codon 245 mutation was 0.82%, compared to 0.4% in Baugh *et al* [52]. Mutation in codon 248 was found in Exon 7, similar to the study of Sisk *et al* [45], with a percentage of 0.82% and contrary to the study of Baugh *et al* [52], where codon 248% was 3.53%. In Exon 8, the mutation was found in codon 283 consistent with the study of Peltonen *et al* [51]. Mutation in codon 175 was found in Exon 5, similar to different studies [51, 52]. All four mutant samples showed only transition mutations contrary to the study of Schneider-Stock *et al* [58] where both transition and transversion mutations were observed. The age of patients was over 45 years old, consistent with the study of Poeta *et al* [14]. In this study with knowledge of the patients’ smoking habits and environmental exposure, we examined *p53* aberrations in primary HNC patients. Three out of the four mutation-containing patients did smoking and were exposed to dust, insecticides and pesticides, similar to the factors observed in the study of Peltonen *et al* [51].

There is a well-established and significant link between smoking and HNC as found in several studies [59–63, 24, 25, 64] and it is known that *p53* aberrations in general play a significant role in human cancers. Though the importance of *p53* in the etiology of HNC has not yet been fully described in the literature, *TP53* mutations in human malignancies have typically been the subject of substantial research. Mutant *p53*, the aberrant protein produced by *TP53* alleles with missense mutations that frequently accumulate in cancer cells, has drawn a great deal of attention [51].

## Conclusion

In the current study, we identified four mutations in exons 5–8 of the *TP53* gene in HNC patients using SSCP. Our findings suggest that *TP53* mutations may have a limited role in the development of HNC in the study population. However, further studies on a large scale are necessary to obtain more detailed results.

## Conflicts of interest

The authors declare no conflict of interest.

## Funding

No source of funding.

## Author contributions

**Summera Fatima:** Data collection, data analysis, result compilation, writing – review, statistical analysis and editing and quality control.

**Asia Bibi:** Conceptualisation, methodology, writing – original draft, supervision.

**Sara Samad Qureshi:** Review, editing and data collection.

**Suman Khan:** Review, editing and data collection.

## Figures and Tables

**Table 1.** Demographic features among cases and controls.

Variable	Cases (*n* = 121) *n* (%)	Control (*n* = 121) *n* (%)	OR	Cl (95%)	*p*- value
Age ≤ 35 >35–55 >55	22 (18.18) 52 (42.97) 40 (50.63)	62 (51.3) 42 (34.7) 17 (14.0)	Reference 3.49 7.79	1.851–6.577 3.726–16.294	<0.001*** <0.001***
Smoking No Yes Smoking frequency 1–10 times/day >10 times/day Smoking duration (years) 1-15 >15	59 (48.7) 62 (51.2) 6 (9.7) 56 (90.3) 16 (25.8) 46 (74.2)	103 (85.1) 18 (14.9) 17 (94.4) 1 (5.6) 17 (94.4) 1 (5.6)	Reference 6.01 Reference 158.6 Reference 48.87	3.25–11.11 17.83–1411 6.01–397.3	<0.001*** <0.001*** <0.001***
Naswar use No Yes Naswar use frequency 1–5 times/day 6–10 times/day Naswar use (years) 1–10 11–20 >20	102 (84.3) 19 (15.7) 6 (31.6) 13 (68.4) 4 (21.1) 5 (26.3) 10 (52.6)	113 (93.4) 8 (6.6) 4 (50.0) 4 (50.0) 4 (50.0) 3 (37.5) 1 (12.5)	Reference 2.63 Reference 2.16 Reference 1.66 0.06	1.10–6.27 0.40–11.74 0.22–12.2 0.83–119.3	0.029* 0.370 0.615 0.069

* *p* ≤ 0.05 = significant;

*** *p* ≤ 0.001 = very highly significant; OR: odds ratio; Cl: Confidence Interval.

*p*-value = Chi-square test

**Table 2.** Non-demographic factors among cases and controls.

Variable	Cases (*n* = 121) *n* (%)	Control (*n* = 121) *n* (%)	OR	Cl (95%)	*p*-value
Oral hygiene Yes No Tooth brush/Miswak use >2 times/day 1 time/day	57 (47.1) 64 (52.9) 19 (33.3) 38 (66.7)	85 (70.2) 36 (29.8) 15 (17.6) 70 (82.4)	Reference 2.82 Reference 0.429	1.64–4.78 0.19–0.938	0.026* 0.034*
Gum bleeding No Yes	105 (86.8) 16 (57.0)	114 (94.2) 7 (5.8)	Reference 2.48	0.98–6.27	0.055
Anemic status No Yes	52 (43.0) 69 (57.0)	116 (95.9) 5 (4.1)	Reference 30.78	11.73–80.7	< 0.001**
Diet Vegetarian Mixed Meat fonder	79 (65.3) 27 (22.3) 15 (12.4)	65 (53.7) 52 (43.0) 4 (3.3)	Reference 0.42 3.08	0.24–0.75 1.03–1.02	0.003* 0.055

* *p* ≤ 0.05 = significant;

** *p* ≤ 0.01 = highly significant; OR: odds ratio; Cl: Confidence Interval; *p*-value = Chi-square test

**Table 3.** Summary of mutant samples.

Serial No.	Exon	Site	Gender	Age	Smoking frequency/day	Occupation	TNM classification	Tumour stage	Tumour grade
1	Exon 5	Larynx	Male	60 years	20/day	Farmer	T3N1M0	III stage	Grade 2
2	Exon 7	Larynx	Male	60 years	20/day	Farmer	T3N1M0	III stage	Grade 2
3	Exon 7	Larynx	Male	45 years	20/day	Shopkeeper	T3N0M0	III stage	Grade 2
4	Exon 8	Oral cavity	Male	60 years	No	Farmer	T2N0M0	I stage	Grade 2

**Table 4.** Spectrum of *TP53* mutations in HNC patients.

Serial No.	Mutation name	Exon	Codon	Type	Event	Genetic alteration	Amino acid alteration
1	Arg175 His	5	175	Transition	G > A	CGC > CAC	Arg > His
2	Gly 245 Ser	7	245	Transition	G > A	GGC > AGC	Gly > Ser
3	Arg 248Gln	7	248	Transition	G> A	CGG > CAG	Arg > Gln
4	Arg 283 His	8	283	Transition	G > A	CGC > CAC	Arg > His
